# A case of hyperlysinemia identified by urine newborn screening

**DOI:** 10.1002/jmd2.12399

**Published:** 2023-10-22

**Authors:** Mehdi Yeganeh, Christiane Auray‐Blais, Bruno Maranda, Amanda Sabovic, Robert J. DeVita, Michael B. Lazarus, Sander M. Houten

**Affiliations:** ^1^ Division of Medical Genetics, Department of Pediatrics, Faculty of Medicine, Centre Hospitalier Universitaire de Québec, Centre Mère‐Enfant Soleil Université Laval Québec City Québec Canada; ^2^ Division of Medical Genetics, Department of Pediatrics, Faculty of Medicine and Health Sciences, Centre de recherche—CIUSSS de l'Estrie‐CHUS Université de Sherbrooke Sherbrooke Québec Canada; ^3^ Department of Pharmacological Sciences Icahn School of Medicine at Mount Sinai New York New York USA; ^4^ Drug Discovery Institute Icahn School of Medicine at Mount Sinai New York New York USA; ^5^ Department of Genetics and Genomic Sciences Icahn School of Medicine at Mount Sinai New York New York USA

**Keywords:** aminoaciduria, ascertainment bias, lysine, newborn screening, sampling bias, substrate reduction therapy

## Abstract

Hyperlysinemia is a rare autosomal recessive deficiency of 2‐aminoadipic semialdehyde synthase (AASS) affecting the initial step in lysine degradation. It is thought to be a benign biochemical abnormality, but reports on cases remain scarce. The description of additional cases, in particular, those identified without ascertainment bias, may help counseling of new cases in the future. It may also help to establish the risks associated with pharmacological inhibition of AASS, a potential therapeutic strategy that is under investigation for other inborn errors of lysine degradation. We describe the identification of a hyperlysinemia case identified in the Provincial Neonatal Urine Screening Program in Sherbrooke, Quebec. This case presented with a profile of cystinuria but with a very high increase in urinary lysine. A diagnosis of hyperlysinemia was confirmed through biochemical testing and the identification of biallelic variants in *AASS*. The p.R146W and p.T371I variants are novel and affect the folding of the lysine‐2‐oxoglutarate domain of AASS. The 11‐month‐old boy is currently doing well without any therapeutic interventions. The identification of this case through newborn urine screening further establishes that hyperlysinemia is a biochemical abnormality with limited clinical consequences and may not require any intervention.

**Synopsis**

A case of hyperlysinemia presenting with a profile of cystinuria in newborn urine screening confirms that hyperlysinema is a biochemical abnormality with limited clinical consequences.

## 1 INTRODUCTION

Hyperlysinemia is a biochemical abnormality caused by 2‐aminoadipic semialdehyde synthase (AASS) deficiency due to mutations in *AASS*.^1^, ^2^, ^3^ AASS is a bifunctional enzyme with lysine 2‐oxoglutarate reductase (LOR) and saccharopine dehydrogenase (SDH) domains.^2^, ^4^ By using NADPH as co‐substrate, the LOR domain of AASS catalyzes the first committed and rate‐limiting step in lysine degradation.^5^, ^6^, ^7^, ^8^, ^9^ Most individuals with *AASS* mutations have hyperlysinemia type I (MIM 238700) due to an isolated LOR defect or combined deficiencies of LOR and SDH. A few cases have been described with an isolated SDH defect, which is characterized by hyperlysinemia in combination with pronounced saccharopinuria (hyperlysinemia type II, MIM 268700). Data from newborn urine screening programs in Massachusetts and New South Wales indicate that hyperlysinemia is an ultra‐rare condition with an estimated frequency of 1 in 300 000–500 000 newborns, respectively.^10^

Hyperlysinemia is generally considered to be a benign metabolic condition. This means that although hyperlysinemia is an inborn error of metabolism that can be diagnosed through biochemical and genetic testing, it is considered non‐harmful to the affected individuals. Several early case reports have associated hyperlysinemia with developmental delay, intellectual disability, and muscle weakness through screening of affected (institutionalized) children for abnormal plasma amino acids.^11^, ^12^ A follow‐up study, however, questioned a causal relationship because healthy hyperlysinemic family members were also identified.^13^ Since then, other reports have been consistent with a benign nature of hyperlysinemia.^14^, ^15^ To formally address the potential pathogenicity of hyperlysinemia without ascertainment bias, Dancis et al.^16^ selected four cases detected in newborn screening programs, four identified in family surveys of a previously diagnosed case, and two affected siblings investigated for short stature. In this study, no adverse effects could be attributed to hyperlysinemia.^16^ Other indirect evidence that points toward the benign nature of hyperlysinemia is available. A child with no clinical manifestations was born to a mother with hyperlysinemia indicating elevated lysine is not teratogenic.^16^ Dietary restriction of lysine was deemed not beneficial in symptomatic cases with the caveat that neurological damage is often irreversible.^1^, ^17^, ^18^ Importantly, the clinical symptoms associated with AASS deficiency are non‐specific, further increasing the probability that ascertainment bias was a factor in the association. This implies that the clinical symptoms in such patients are caused by other genetic or environmental factors. Indeed, in two (related) cases, we found a novel contiguous gene deletion syndrome involving *AASS* and *PTPRZ1*. Loss‐of‐function of *PTPRZ1* was likely responsible for the severe neurological disease phenotype in these cases.^1^ In another case, prenatal toxic exposures were suspected.^1^ Studies using mouse models confirm that hyperlysinemia type I is benign^19^, ^20^ but also indicate that hyperlysinemia type II (i.e., with saccharopinuria) is a potentially harmful condition.^20^, ^21^

Although reasonably clear and generally accepted, one can never make the unequivocal statement that hyperlysinemia is entirely benign. Further study of cases with hyperlysinemia is warranted for two important reasons. First, cases with hyperlysinemia due to AASS deficiency continue to be diagnosed. With only relatively few studies available, counseling can remain challenging in particular in light of a potential clinical difference between type I and type II hyperlysinemia. Second, substrate reduction through inhibition of the LOR domain of AASS is being investigated as a novel treatment option for glutaric aciduria type 1 (GA1; MIM 231670) and pyridoxine‐dependent epilepsy caused by mutations in *ALDH7A1* (PDE‐ALDH7A1; MIM 266100).^19^, ^22^, ^23^, ^24^ In order to collect data that can further de‐risk inhibition of AASS as a potential therapeutic approach for GA1 and PDE‐ALDH7A1, we are actively collecting information on cases of hyperlysinemia. Here, we report the identification of a hyperlysinemia case detected by the Provincial Neonatal Urine Screening Program in Sherbrooke, Quebec, Canada.

## 2 CASE REPORT

Our case is an 11‐month‐old boy. He was born at term following an uncomplicated pregnancy with normal growth parameters. The perinatal period was also normal. Since birth, he has been regularly followed by his pediatrician and our center with normal growth, development, and physical exam. Newborn screening by dried blood spot was negative. The province of Quebec has the only newborn screening program that employs urine screening in addition to dried blood spot screening. This program has over 50 years of experience.^25^, ^26^, ^27^, ^28^ Briefly, newborn urine samples are collected by parents at home at 21 days of age (compliance rate of ~90% over the years) and sent by regular mail to Sherbrooke. Samples are processed and analyzed by thin‐layer chromatography. After quantitation, abnormal samples are referred to one of four medical centers in the Province of Quebec for diagnostic confirmation. The urine screen of this case showed a profile of cystinuria at screening of the 21‐day‐old urine filter paper sample, but with a very high increase in urinary lysine (in mmol/mol creatinine: Cystine, 96; Orn, 89; Lys, 3810; Arg, 124; creatinine, 0.16 mM). Upon referral at 53 days of age, urine lysine was 5541 mmol/mol creatinine with parallel increases of cystine, ornithine, arginine, citrulline, homocitrulline and cystathionine (Table S1). The plasma lysine concentration was 1222 μmol/L (Table S2). Saccharopine was undetectable in the urine. Urinary pipecolic acid was just above the upper normal limit at 0.8 mmol/mol creatinine (0.01–0.7). The dibasic aminoaciduria associated with hyperlysinemia has been described before and is likely caused by competitive inhibition of the dibasic amino acid transporters SLC7A7 and SLC7A9/SLC3A1 in the proximal tubule of the kidney by the high levels of lysine in the pre‐urine.^14^, ^15^, ^19^, ^29^

Molecular testing revealed biallelic variants in *AASS*, c.436C>T (p.R146W) and c.1112C>T (p.T371I) with the father and mother heterozygous for p.R146W and p.T371I, respectively. Molecular testing was negative for variants in other genes that may be associated with lysinuria/hyperlysinemia including *ARG1*, *SLC7A7*, *SLC7A9*, *SLC3A1*, and *NADK2*. The uncertain clinical significance of hyperlysinemia was discussed with the parents. The parents opted for not starting him on a lysine‐restricted diet. Parents do not report any persistent or intermittent neurological symptoms, nor emergency visits. Complete urine and plasma amino acid profiles at 53 days and 11 months of age are provided in Tables S1 and S2.

## 3 THE MOLECULAR BASIS OF AASS DEFICIENCY IN THE CURRENT CASE

**FIGURE 1:**
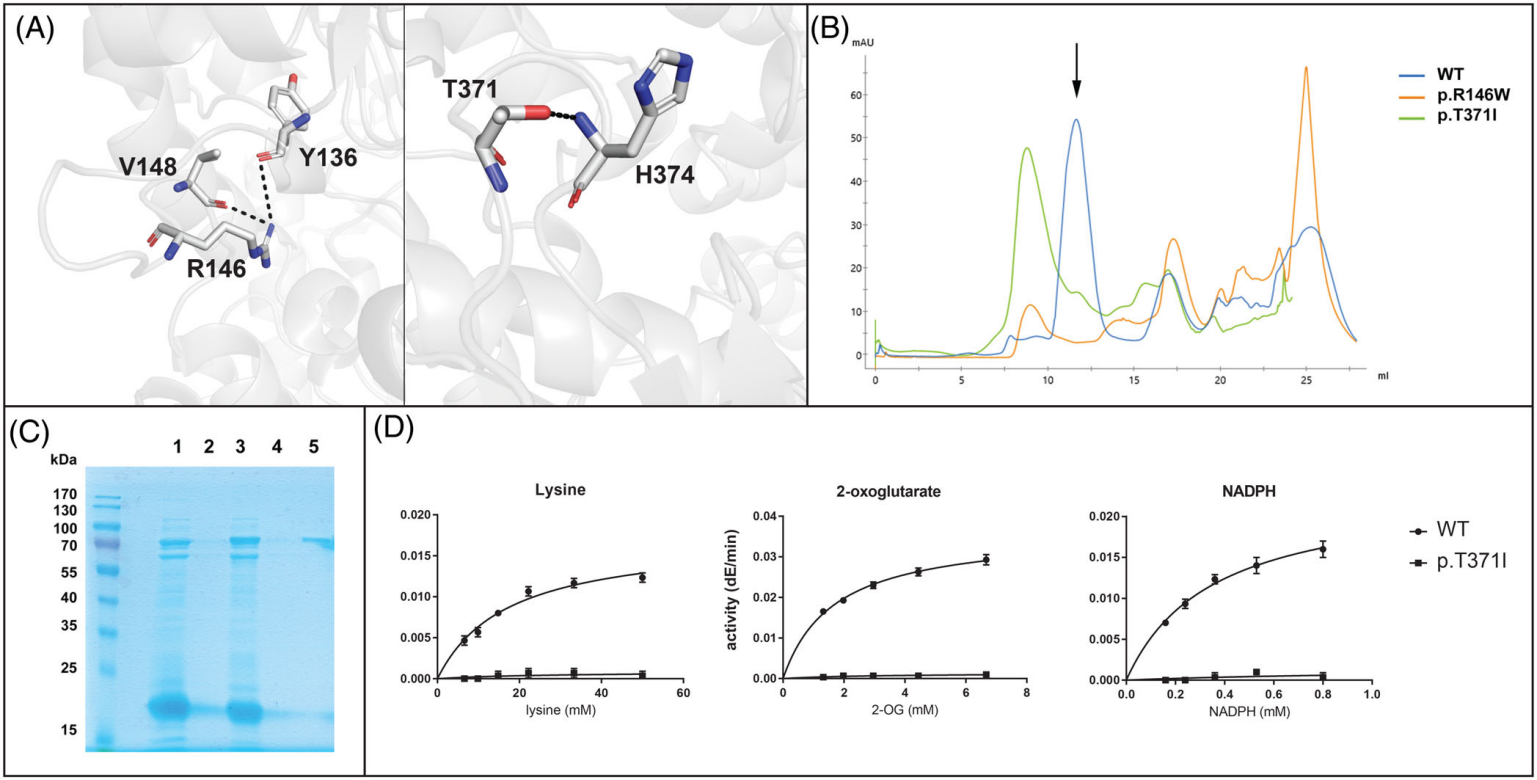
The molecular basis of AASS deficiency in the current case. (A) Structural analysis of LOR patient variants. Left panel shows contacts to R146 with potential hydrogen bonds shown with dashed lines. Right panel shows contacts to T371. PDB code for the LOR structure is 8E8U.^6^ (B) FPLC traces of LOR variants. The UV trace for each variant on the Supderdex200 Increase column is shown overlaid along with the WT, with the variant identified in the inset. An arrow indicates where the soluble protein elutes. (C) Purification of LOR variants. The different variants were purified and analyzed by Coomassie staining of an SDS‐PAGE gel. Lanes 1–2: p.R146W nickel elution, gel filtration aggregation peak. No soluble peak was observed for this variant. Lanes 3–5: p.T371I nickel elution, aggregation peak, soluble peak of the expected size. Note that the SUMOstar tag migrates at ~15‐20 kDa. Calculated MW of the His‐SUMOstar‐LOR monomer is 62.4 kD. (D) LOR activity. LOR activity with variable concentrations for each of the three LOR substrates. The concentrations for the varied substrates are indicated in the graph. Fixed substrate concentrations are 15 mM L‐lysine, 1 mM 2‐OG, and 0.3 mM NADPH. WT protein was tested at 96 ng per 200 μL reaction, p.T371I was tested at 167 ng per 200 μL reaction.

Both *AASS* variants are novel, affect conserved amino acids in the LOR domain, have very low allele frequencies in gnomAD, and are predicted to be damaging/deleterious. The CADD/PHRED scores are 32 for c.436C>T and 25.1 for c.1112C>T. These in silico analyses all indicate that the identified *AASS* variants are deleterious and thus causal for hyperlysinemia in this case. Using our recently solved LOR structure, we can better understand the consequences of these amino acid substitutions.^6^ Arg 146 forms two potential hydrogen bond interactions with backbone carbonyls, from Tyr 136 and Val 148 (Figure 1A). Therefore, it is possible that this residue is required for stabilizing the fold of the protein, and its mutation destabilizes the protein. Thr 371, similarly, forms a potential hydrogen bond with backbone amide nitrogen of His 374, which forms a turn (Figure 1A). In order to confirm the pathogenicity of the identified *AASS* variants, we expressed the variant proteins as recombinant isolated LOR protein in *E. coli*.^6^ We obtained lower yields for both proteins than for the wild‐type protein. We then purified the variants by size exclusion chromatography to determine if any of the protein was folded at the expected tetrameric size or was in the aggregation peak (Figure 1B,C). The W146 variant was largely insoluble and we were unable to obtain any protein at the correct elution volume to analyze for enzyme activity. The I371 variant was insoluble, but there was a small peak at the correct size (Figure 1B,C). This I371 variant protein, however, was inactive even at the highest tested (near saturating) concentration of the LOR substrates lysine, 2‐oxoglutarate, and NADPH (Figure 1D). We conclude that both mutations prevent proper folding of the LOR protein. Several other *AASS* variants also have been observed to affect protein stability.^1^, ^20^

## 4 DISCUSSION

Herein we present a case of hyperlysinemia identified through newborn urine screening in Sherbrooke, Quebec. We identified two damaging variants in the LOR domain of AASS, which is consistent with the diagnosis of hyperlysinemia type I (i.e., without saccharopinuria). This case was the first detected over the last 6 years in the province of Quebec giving an estimated incidence of ~1 in 411 000 newborns, which supports previous findings that hyperlysinemia is an ultra‐rare condition.^10^ This case is 11 months of age and has been asymptomatic, which is consistent with the notion that hyperlysinemia is benign, but mild consequences of his biochemical disorder cannot be completely ruled out yet. Our results also do not provide any additional information on the consequences of lifelong inhibition of AASS. In order to further address this issue, it is possible to study previously diagnosed cases. This approach, however, may be affected by ascertainment bias. Alternatively, one may try a “genome‐first” approach and identify *AASS* KO or hypomorphic individuals based on the presence of biallelic deleterious variants. This is challenging since hyperlysinemia is an ultra‐rare condition in presumably healthy people and most of the missense variants are of unknown significance. One potential solution for this barrier is to study cohorts with substantial autozygosity. Using this approach a healthy adult with a rare *HAO1* (glycolate oxidase) knockout was recently described to support a novel substrate reduction therapeutic strategy for primary hyperoxaluria.^30^ We conclude that despite its likely benign nature, hyperlysinemia is an interesting condition that should be further studied.

## 5 MATERIALS AND METHODS

### 5.1 Expression and analysis of variant LOR proteins

AASS LOR variants were purified as uncut SUMOstar‐LOR protein as reported previously.^6^ Briefly, His‐SUMOstar‐LOR [residues 21–470] constructs were expressed in *E. coli* LOBSTR cells and purified with IMAC chromatography. The elution was then purified further by size‐exclusion chromatography (Superdex200 Increase) on an AKTA FPLC (Fast Protein Liquid Chromatography) system. This enabled separation of unfolded and folded states of the protein. They were then analyzed on an 11% polyacrylamide gel and stained with Coomassie. LOR activity was measured spectrophotometrically at 340 nm by using 2‐oxoglutarate, lysine, and NADPH as substrates.^6^

## FUNDING INFORMATION

Research reported in this publication was supported by the Eunice Kennedy Shriver National Institute of Child Health & Human Development and the National Institute of General Medical Sciences of the National Institutes of Health under Award Numbers R01 HD112518 (to Sander M. Houten, Robert J. DeVita, and Michael B. Lazarus), R21 HD102745 (to Sander M. Houten and Robert J. DeVita), and R35 GM124838 (to Michael B. Lazarus). The content is solely the responsibility of the authors and does not necessarily represent the official views of the National Institutes of Health.

## CONFLICT OF INTEREST STATEMENT

Sander M. Houten reports grants from NIH/Eunice Kennedy Shriver National Institute Of Child Health & Human Development during the conduct of the study. Robert J. DeVita reports grants from NIH/Eunice Kennedy Shriver National Institute Of Child Health & Human Development during the conduct of the study. Michael Lazarus reports grants from NIH/Eunice Kennedy Shriver National Institute Of Child Health & Human Development and NIH/National Institute of General Medical Sciences during the conduct of the study. The remaining authors declare no conflicts of interest.

## ETHICS STATEMENT

Ethical considerations were respected in accordance with the Canadian Tri‐Council Policy Statement: Ethical Conduct for Research Involving Humans—TCPS 2 (2022). Consent for publication was given by the mother of the presented case.

## Supporting information


**Table S1.** Urine amino acid profile.
**Table S2.** Plasma amino acid profile.

## Data Availability

Tables S1 and S2 are available on the Journal's website. No other archived datasets are associated with this article.
