# Supplementary material for: A case of hyperlysinemia identified by urine newborn screening
Source: JIMD Rep. 2023 Oct 22;64(6):440–5. doi: 10.1002/jmd2.12399 (PMC10623103; doi:10.1002/jmd2.12399)
Supplement: Supplementary file 1 — Table S1. Urine amino acid profile. Table S2. Plasma amino acid profile. [file JMD2-64-440-s001.docx]

Supplemental table 1: Urine amino acid profile.

|  | 53 days old  mmol/mol creatinine  (reference 0-5 month-old) | 11 months old  mmol/mol creatinine  (reference 5-24 month-old) |
| --- | --- | --- |
| **Taurine** | **835 (30-616) H** | **25 (11-463)** |
| Aspartic acid | 32 (11-61) | 0 (<6) |
| Hydroxyproline | 230 (0-332) | 4 (1-31) |
| **Threonine** | **461 (18-143) H** | **43 (23-79)** |
| **Serine** | **376 (76-288) H** | **85 (66-186)** |
| Asparagine | 98 (10-107) | 16 (12-60) |
| Glutamic acid | 36 (0-32) H | 5 (4-13) |
| Glutamine | 610 (46-293) H | 168 (84-261) |
| Sarcosine | 0 (0) | 0 (<5) |
| Aminoadipic acid | 78 (0-73) | 20 (5-37) |
| Proline | 141 (0-146) | 5 (3-18) |
| Glycine | 1402 (188-1390) H | 294 (107-678) |
| Alanine | 366 (51-350) H | 81 (40-182) |
| **Citrulline** | **42 (0-17) H** | **50 (<3) H** |
| Valine | 37 (0-41) | 12 (9-23) |
| **Cystine** | **110 (4-39) H** | **64 (5-16) H** |
| Methionine | 0 (0) | 8 (<4) H |
| **Homocitrulline** | **31 (0-41)** | **207 (1-12) H** |
| Alloisoleucine | 0 (0) | 0 (<2) |
| **Cystathionine** | **93 (0-32) H** | **7 (<4) H** |
| Isoleucine | 7 (0-14) | 1 (3-7) |
| Leucine | 64 (0-24) H | 17 (6-16) |
| **Tyrosine** | **53 (9-55)** | **61 (11-50) H** |
| Phenylalanine | 23 (3-37) | 35 (9-29) H |
| Homocystine | 44 (0-3) H | 0 (0) |
| **Ornithine** | **63 (0-48) H** | **36 (<6) H** |
| **Lysine** | **5541 (21-243) H** | **11283 (8-78) H** |
| Hydroxylysine | N/A | 14 (<6) H |
| Histidine | 636 (58-324) H | 355 (82-366) |
| **Arginine** | **133 (0-31) H** | **190 (2-10)** |
| Tryptophan | N/A | 23 (7-29) |

N/A, not analyzed

Supplemental table 2: Plasma amino acid profile.

|  | 53 days old  µmol/L  (Reference 0-5 month-old) | 11 months old  µmol/L  (Reference 5-24 month-old) |
| --- | --- | --- |
| Taurine | 130 (41-217) | 65 (42-146) |
| Aspartic acid | 15 (6-36) | 26 (5-40) |
| Hydroxyproline | 41 (0-53) | 12 (9-29) |
| **Threonine** | **347 (75-297) H** | **106 (63-209)** |
| Serine | 148 (86-298) | 126 (113-209) |
| Asparagine | 88 (27-84) | 44 (29-67) |
| Glutamic acid | 64 (47-221) | 58 (36-126) |
| Glutamine | 659 (229-884) | 390 (405-690) |
| Sarcosine | 0 (0) | 2 (1-3) |
| Aminoadipic acid | 8 (0-8) | N/A |
| Proline | 181 (91-321) | 188 (105-287) |
| Glycine | 260 (144-462) | 177 (127-358) |
| Alanine | 302 (127-447) | 271 (172-525) |
| Citrulline | 28 (6-35) | 16 (10-36) |
| Valine | 228 (84-325) | 231 (137-324) |
| Cystine | 15 (2-36) | 3 (2-22) |
| **Methionine** | **63 (13-43) H** | **28 (13-37)** |
| **Homocitrulline** | **0 (0)** | **4 (0) H** |
| Alloisoleucine | 0 (0) | 1 (<2) |
| Cystathionine | 0 (0-3) | 0 (0) |
| Isoleucine | 67 (12-104) | 56 (40-112) |
| Leucine | 121 (40-179) | 125 (73-205) |
| Tyrosine | 67 (27-103) | 58 (37-116) |
| Phenylalanine | 38 (30-84) | 67 (44-95) |
| Homocystine | 0 (0-1) | 0 (0-1) |
| Ornithine | 91 (33-235) | 31 (28-106) |
| **Lysine** | **1222 (59-295) H** | **1232 (90-227) H** |
| Histidine | 110 (45-112) | 77 (55-101) |
| Arginine | 124 (14-111) | 103 (56-143) |
| Tryptophan | N/A | 73 (27-86) |

N/A, not analyzed
